# Impregnation of poly(L-lactide-*ran*-*δ*-valerolactone) with essential bark oil using supercritical carbon dioxide

**DOI:** 10.1038/s41598-019-52910-2

**Published:** 2019-11-08

**Authors:** Chikara Tsutsumi, Souta Manabe, Susumu Nakayama, Yuushou Nakayama, Takeshi Shiono

**Affiliations:** 1 Department of Applied Chemistry and Biotechnology, National Institute of Technology (KOSEN), Niihama College, Niihama, Japan; 2 Department of Applied Chemistry, Graduate School of Engineering, Hiroshima University, Hiroshima, Japan

**Keywords:** Environmental sciences, Astrochemistry, Materials science

## Abstract

This work studied the incorporation of essential bark oil from *Thujopsis dolabrata* var. *hondae*, which is known to repel various insects, in poly(L-lactide-*ran*-*δ*-valerolactone) [poly(L-LA-*ran*-VL)] using supercritical carbon dioxide (scCO_2_). The poly(L-LA-*ran*-VL) was synthesized by first purifying the monomers by azeotropic distillation with benzene, followed by polymerization with Sn(oct)_2_ using the same equipment, representing an efficient one-pot process. The copolymerization of L-LA with VL using this technique at a feed ratio of 90/10 mol/mol gave poly(L-LA-*ran*-VL) (91/9) with a molecular weight of 6.48 × 10^4^ g/mol and a high yield of 74.9%. Products with molecular weights over 5.0 × 10^4^ g/mol were obtained at L-LA feed proportions of 70 to 90%. Impregnation trials were conducted between 40 and 120 °C at 14 MPa for 3 h. The oil content of a 73/27 specimen was found to increase significantly during processing at 100 or 120 °C. During enzymatic degradation with proteinase K, the 91/9 specimen showed the fastest degradation rate. Although the 71/29 sample was slowly hydrolyzed in a phosphate buffer at pH 7.0, the release of oil vapor from this material was slightly higher than that from the 91/9 specimen, and the vapor release rate continuously increased throughout the hydrolysis process.

## Introduction

At present, various issues represent major challenges to human welfare in modern society, including food poisoning caused by bacilli, health hazards due to mold exposure, and insect and wildlife damage to farming operations. The latter is especially serious because, combined with climate change and loss of arable land, this affects the production and quality of agricultural products^1–5^. Damage to buildings by termites is also problematic and has been reported to lead to the collapse of residences in response to earthquakes^6,7^. There have been various attempts to address these issues, including the development of insect repellents^8,9^. Both insect repellents and insecticides are currently available, and there are various reports evaluating the efficacies and toxicities of chemical and plant-derived insect repellents^10^. Examples include citronellal, which is the main component of citronellal oil^11^, and eucalyptus, which is an important essential oil used extensively in the food, perfume and pharmaceutical industries. This oil has anti-microbial, fungicidal, insect repellent, herbicidal, acaricidal and nematicidal properties, and so is used to protect against bacteria, fungi, insects, nematodes, weeds and mites as an environmentally-benign pest control agent^12^. As another example, essential oil from the bark of *Thujopsis dolabrata* var. *hondae* is known to be highly toxic to *Reticulitermes speratus*, which is found widely throughout Japan. This oil additionally has an antibacterial effect and is employed for deodorization as well. The use of such essential oils in place of conventional chemical agents can be desirable, although it is necessary to suppress the release of vapors from the highly volatile oils, possibly by using controlled-release mechanisms such as those found in drug delivery systems (DDSs)^13–15^.

Our group has researched advanced controlled-release materials for DDSs in conjunction with the use of supercritical carbon dioxide (scCO_2_). Because carbon dioxide is a nontoxic fluid with a relatively low critical point (*T*_c_ = 31.1 °C, *P*_c_ = 7.376 MPa), it is widely used as a supercritical fluid in the food^16,17^, pharmaceutical^18,19^, chemical^20,21^ and plastics industries^22,23^, and is often referred to as a “green” solvent. scCO_2_ is also a good solvent for the dissolution and extraction of organic compounds and so is well-suited to the dissolution of essential oils and the plasticization of biodegradable polymers acting as controlled-release materials. Thus, scCO_2_ can be used to combine these materials at low temperatures to produce composites based on the incorporation of oils in biodegradable polymers, with the aim of obtaining the spontaneous release of the oil vapors when required. To date, this technique has been applied to encapsulate oils in poly(L-lactide) [poly(L-LA)] or copolymers consisting of L-LA with other cyclic compounds^24–27^. Such technology could result in minimal environmental impact as well as lower energy usage and a reduction in labor.

Because poly(L-LA) is not readily impregnated with many oils, random copolymers of L-LA with *ε*-caprolactone (CL), 2,2-dimethyl trimethylenecarbonate (2,2-DTMC), tetramethylene carbonate (TEMC) or 1,5-dioxepan-2-one (DXO) have been synthesized using tin 2-ethyl-hexanoate (Sn(oct)_2_) in an attempt to the increase the oil content of the polymer^24–27^. Although lowering the melting point or heat of fusion (*T*_m_ or Δ*H*_m_) of the polymer has been found to increase the oil uptake, even films with high L-LA proportions tend to melt during scCO_2_ processing at the relatively low temperature of 60 °C. The synthesis of these copolymers via the ring-opening polymerization of seven-membered cyclic compounds together with L-LA generates either ester or carbonate bonds. Because the distance between these bonds is long, the flexibility of the polymer chain is increased, which in turn reduces both the *T*_m_ and Δ*H*_m_ of the copolymer. In contrast, the copolymerization of L-LA with *δ*-valerolactone (VL), which is a six-membered cyclic compound, increases the *T*_m_ and Δ*H*_m_ of the L-LA copolymer and inhibits melting, thus increasing the amount of essential oil included in the polymer when using scCO_2_. Monomers such as L-LA and VL require purification by recrystallization, sublimation or distillation prior to polymerization and so, in this work, the monomers were purified by azeotropic distillation^28–31^ with benzene. In addition, an efficient one-pot process within the same equipment was devised, and the resulting degree of polymerization was evaluated.

The aim of this study was to impregnate essential bark oil (which is an effective insect repellant) into poly(L-LA-*ran*-VL) at high concentrations using scCO_2_, to obtain a long-acting controlled-release repellent. The extent of impregnation of the essential oil into synthetic poly(L-LA-*ran*-VL) films was therefore assessed, and the effects of the copolymer composition and processing temperature were investigated. The release of the essential oil following impregnation of the films was also examined by monitoring the release of the volatile compound during the hydrolysis of various samples.

## Results and Discussion

### Synthesis of poly(L-lactide-*ran*-*δ*-valerolactone)

In a previous study, CL, TEMC and DXO, representing seven-membered cyclic esters and a carbonate, were copolymerized with L-LA, and the incorporation of essential bark oil into the resulting materials was evaluated^24^. The incorporation of CL, TEMC or DXO introduced long methylene chains into the main polymer chain, thus lowering *T*_m_ and *T*_g_ relative to those of pure poly(L-LA). Because the crystallinity was also reduced considerably, partial melting of the surface during processing with scCO_2_ was observed even at low temperatures.

**Figure 1:**
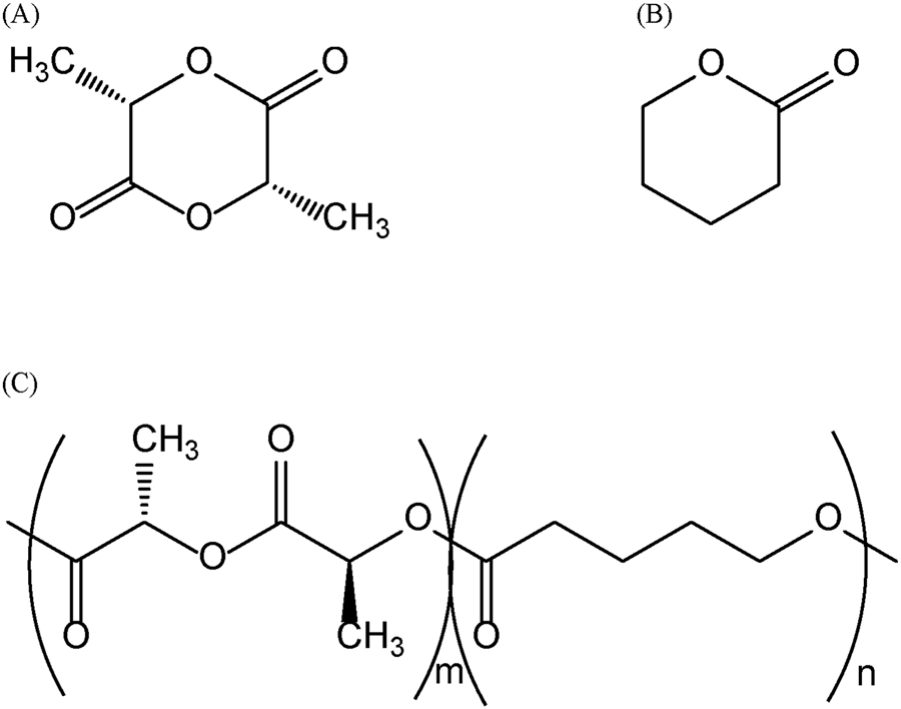
Chemical structures of (**A**) L-lactide (L-LA), (**B**) *δ*-valerolactone (VL) and (**C**) poly(L-lactide-*ran*-*δ*-valerolactone) (poly(L-LA-*ran*-VL)).

In the present work, the copolymer was synthesized using VL (Fig. 1), which is a six-membered cyclic ester with a shorter methylene chain, and the impregnation of the copolymer with the oil using scCO_2_ was evaluated. In order to obtain a high molecular weight polymer in conjunction with a high yield, it is typically necessary to first purify the monomers and to perform the polymerization under an inert gas, such as argon or nitrogen. In this work, an efficient synthetic method was developed so as to simplify the process. Therefore, azeotropic distillation, which is often applied to the refining of monomers^28–31^ and manufacture of alcohol, was used. The monomer was purified by removing trace amounts of water and polymerization was carried out using a single standard distillation apparatus.

**Figure 2:**
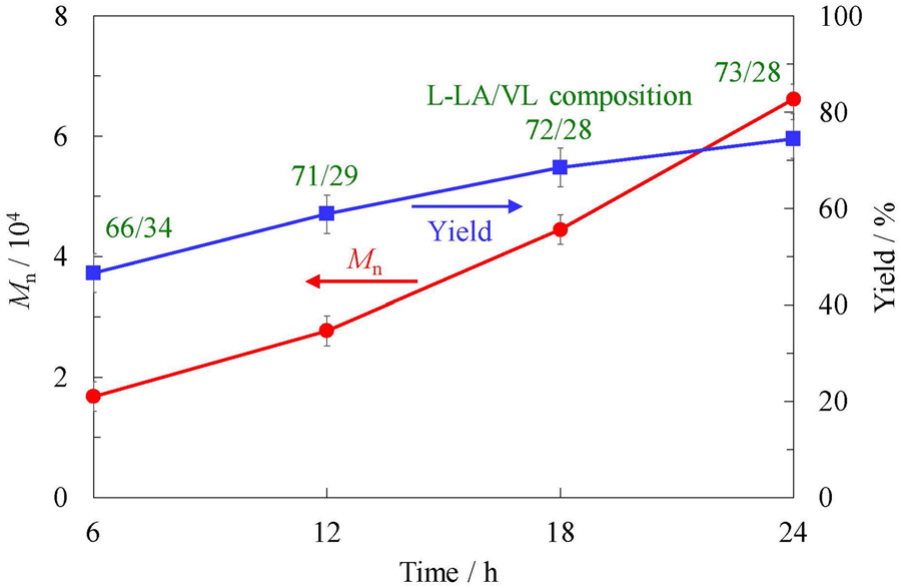
Molecular weight and yield obtained by copolymerization of L-LA and VL at 70/30 of feed ratio using Sn(oct)_2_ in benzene/ethylbenzene (10:8) at 150 °C as functions of reaction time.

While developing the copolymerization method, the effect of the polymerization time was initially studied. These trials were performed using a 70/30 mol/mol of L-LA/VL feed ratio at 150 °C, and the resulting yields, molecular weights and L-LA/VL proportions in the product were evaluated after 6, 12, 18 and 24 h. The results are shown in Fig. 2. A copolymer synthesis performed for 6 h was found to produce a 47% yield and a molecular weight of 1.68 × 10^4^ g/mol, together with an L-LA/VL ratio in the polymer of 66/34. The yield, molecular weight and L-LA proportion all increased as the polymerization time was extended.

**Figure 3:**
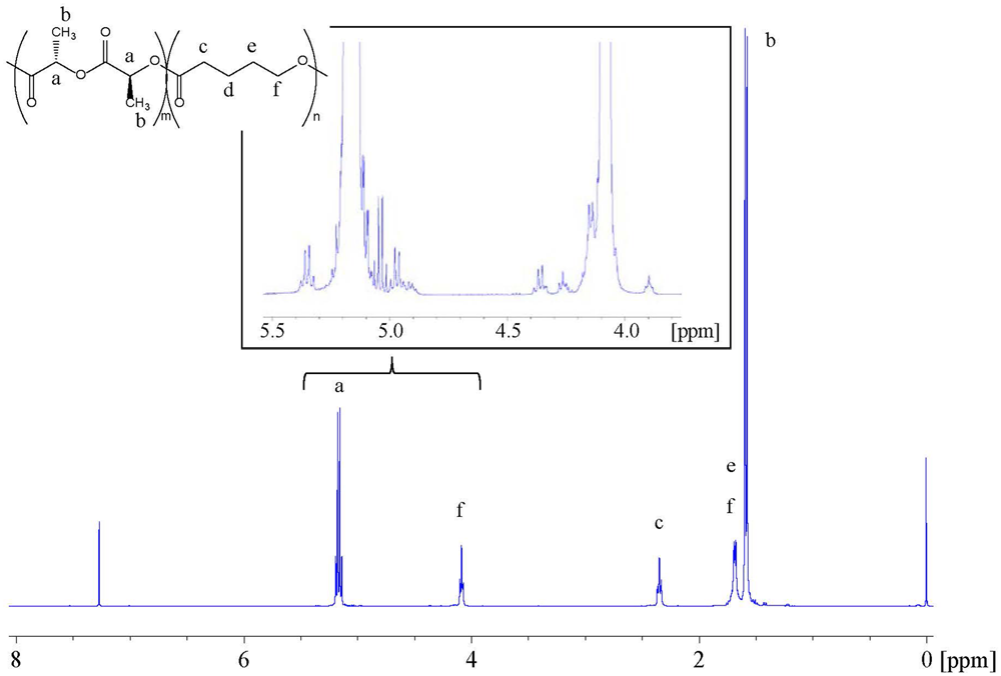
^1^H NMR spectrum of poly(L-LA-*ran*-VL) (73/27) specimen.

**Figure 4:**
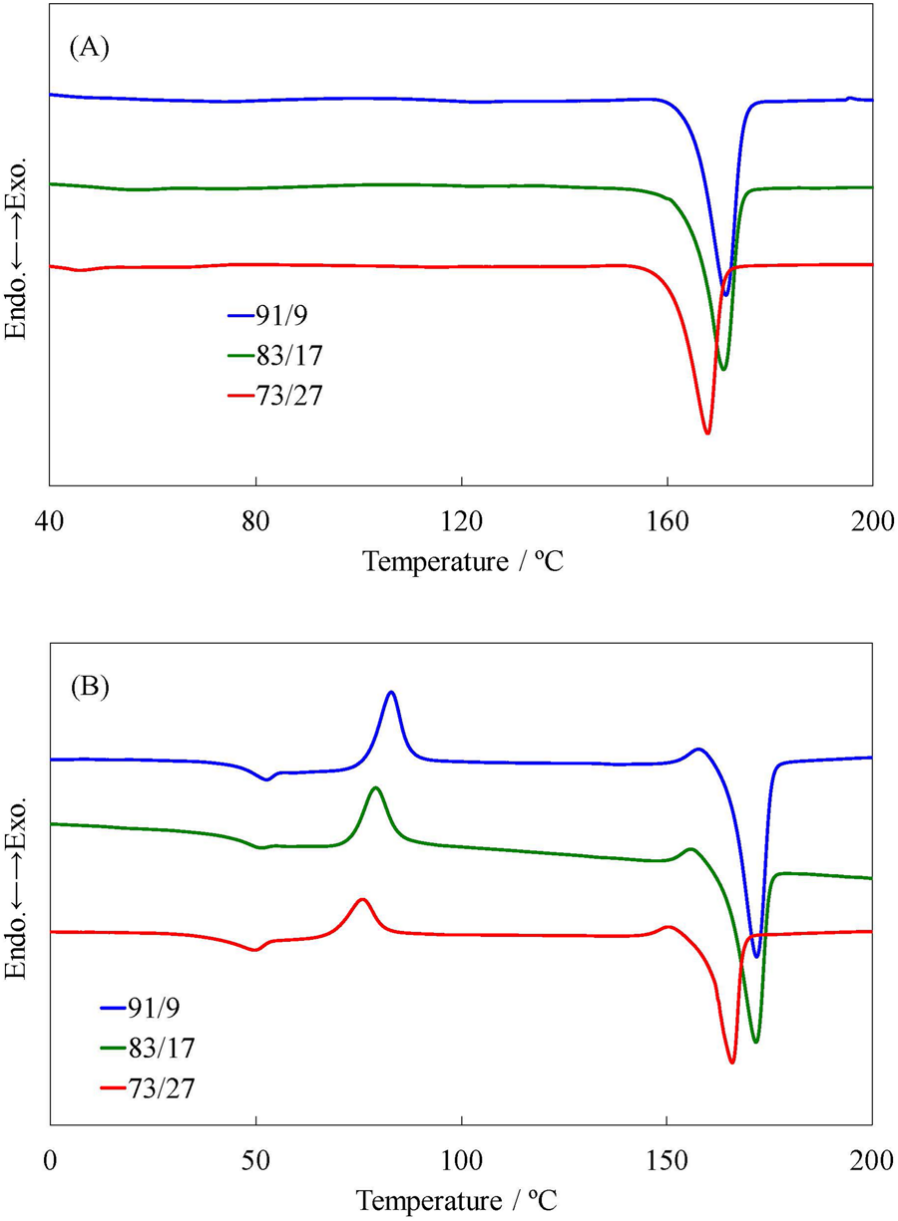
DSC thermograms of poly(L-LA-*ran*-VL) specimens acquired during (**A**) first and (**B**) second heating step.

**Figure 5:**
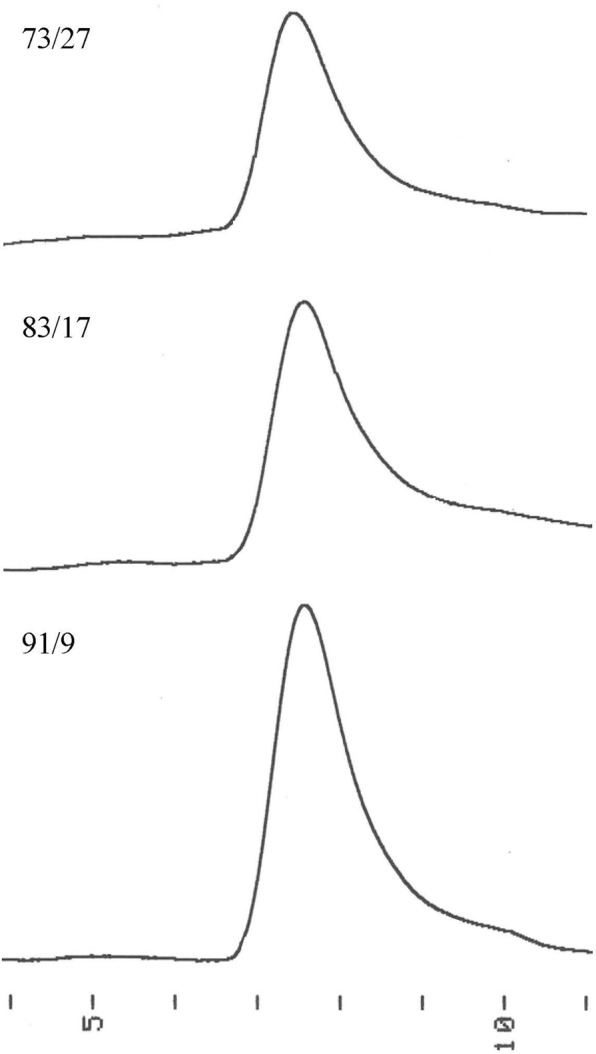
GPC data for poly(L-LA-*ran*-VL) specimens.

Table 1 summarizes the results of copolymerization at 150 °C for 24 h. A ^1^H nuclear magnetic resonance (NMR) spectrum of the poly(L-LA-*ran*-VL) specimen having a 73/27 composition is shown in Fig. 3, while differential scanning calorimetry (DSC) thermograms and gel permeation chromatography (GPC) data obtained from the synthesized poly(L-LA-*ran*-VL) specimens are presented in Figs 4 and 5, respectively. Copolymerization of L-LA with VL resulted in poly(L-LA-*ran*-VL) having a molecular weight of 6.48 × 10^4^ g/mol at a high yield of 74.9% in the case of the specimen with a monomer ratio of 91/9. Thus, the composition of the copolymer was almost the same as the feed ratio. It has been reported that the copolymerization of L-LA with CL at a 90/10 feed ratio gives a similar molecular weight of 7.06 × 10^4^ g/mol and a composition of 92/8^24^. The *T*_m_, Δ*H*_m_ and *T*_g_ values for the poly(L-LA-*ran*-CL) were found to be 154.2 °C, 30.9 J/g and 49.8 °C, respectively. Although the *T*_g_ of the poly(L-LA-*ran*-VL) (91/9) specimen in the current work was almost equal to that of the poly(L-LA-*ran*-CL) (with a value of 50.3 °C), its *T*_m_ and Δ*H*_m_ were considerably higher, at 173 °C and 39.2 J/g, respectively, indicating a highly crystalline polymer. As a result of the shorter methylene lengths compared to those obtained with CL, the flexibility of the polymer chain was suppressed and thus the material crystallized more readily. The poly(L-LA-*ran*-VL) (73/27) also had a relatively high molecular weight, despite the lower L-LA content, and its *T*_m_ and Δ*H*_m_ were decreased only slightly compared to that of the poly(L-LA). These results suggest that the incorporation of VL units promotes crystallization of the polymer chain, and the copolymer with an L-LA content over 70% was used for the impregnation experiments. As demonstrated by the ^1^H NMR and DSC data, random copolymerization of the VL and L-LA units thus modified the thermal characteristics of the poly(L-LA), such as the *T*_m_ and *T*_g_, while reducing the crystallinity of the product. The random copolymer is therefore likely to hold an increased quantity of essential oil. The copolymerization process was also found to generate a material in which the proportions of L-LA and VL were almost equal to those in the raw material mixture. Furthermore, a high degree of polymerization was obtained from this efficient one-pot process, which also served to purify the monomer.

**Table 1:** Reaction conditions and results for ring-opening copolymerization of L-LA and VL catalyzed by Sn(oct)_2_^a^.

Feed [mol/mol]	Solvent/Monomer [ml/g]	Yield [%]	*M*_n_/10^4b^ [g/mol]	*M*_w_/*M*_n_^b^	*T*_m_^c^ [°C]	Δ*H*_m_^c^ [J/g]	*T*_g_^c^ [°C]	Comp.^d^ [mol/mol]
90/10	14.0	74.9	6.48	1.63	173.1	39.2	48.4	91/9
80/20	14.0	63.2	5.13	1.62	172.8	35.4	43.0	83/17
70/30	14.0	74.5	6.62	1.55	166.9	34.8	37.8	73/27

^a^Copolymerization of L-LA with VL was carried out in mixed solvent of benzene and ethylbenzene in a ratio of 10:4 at a [monomer]/[Sn(oct)_2_] ratio of 1000 mol/mol at 150 °C for 24 h. ^b^Determined by GPC. ^c^Determined by DSC. ^d^Determined by ^1^H NMR. Comp.: Composition.

### Impregnation of poly(L-LA-*ran*-*δ*-valerolactone) with essential bark oil using supercritical carbon dioxide

**Figure 6:**
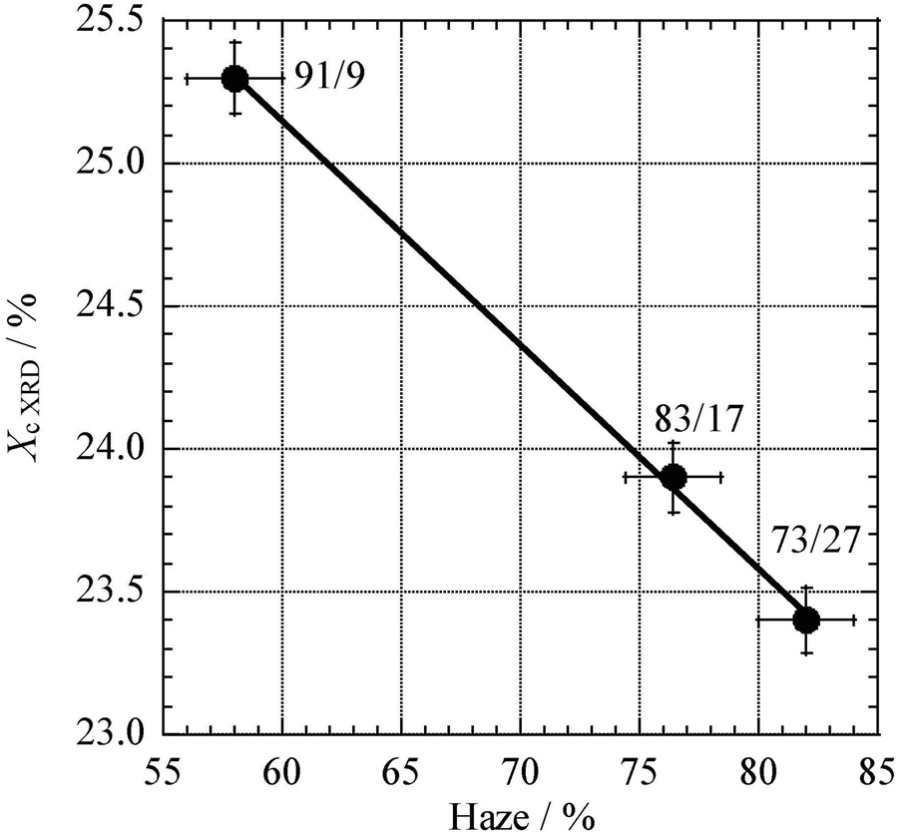
*X*_c XRD_ values for L-LA/VL random copolymer films as a function of haze values.

In prior work, poly(L-LA) random copolymers were impregnated with essential bark oil using scCO_2_, while varying processing conditions such as the pressure, temperature and time^24^. The oil could be most efficiently impregnated into the copolymer at a processing pressure of 14 MPa, and a time span of 3 h was most suitable. Thus, the present impregnation experiments were also conducted at 14 MPa and 3 h, over the temperature range from 40 to 120 °C. The physical properties of the copolymer films used for the impregnation experiments are shown in Table 2. To allow comparisons, pure poly(L-LA) and poly(VL) were also assessed, and the resulting physical properties are provided in Table 3. The thermal properties (*T*_m_, *T*_g_, Δ*H*_m_), degree of crystallinity (*X*_c XRD_) and haze were all determined using 100 μm thick films produced by the solvent-casting method. The haze of a film is a measure of the degree to which it appears cloudy or translucent. If a polymeric film has a high degree of crystallinity, it will tend to be more cloudy. In this work, the cloudiness of each film (as determined by a haze meter) was considered to equal its haze value. The poly(L-LA-*ran*-VL) (91/9) had the highest Δ*H*_m_ (39.2 J/g), along with *X*_c XRD_ and haze values of 25.3% and 58.0%, respectively. The haze values were found to increase as the L-LA content was reduced, while *X*_c XRD_ was decreased. The parameters *T*_p_ and *T*_d_, both determined using the haze meter, indicate the degrees of parallel and diffuse light transmittance, respectively, and are correlated with the arrangement and state of the polymer molecules in the specimen. *T*_d_ is determined by subtracting *T*_p_ from the total light transmittance (*T*_t_), which is equal to the incoming light. These values can be converted into haze readings, which are typically used to assess the crystallinity of a polymer. Specifically, a high haze value indicates a cloudy sample in which light undergoes diffusion, and is a measure of the transparency of the material. The *T*_p_ values also decreased as the L-LA proportion was lowered, likely due to increases in the relative amounts of amorphous regions that did not transmit parallel light. The relationship between the *X*_c XRD_ and the haze value was examined by plotting these data (Fig. 6). In the case of the copolymers having composition ratios of 91/9 to 73/27, the *X*_c XRD_ and the haze value exhibit a very high degree of correlation with an almost linear relationship. Thus, if the films are prepared while keeping the solution concentration, temperature and drying conditions constant, a linear relationship is obtained. At high L-LA proportions, the degree of crystallinity was high but the haze value was reduced, while the opposite was true at lower L-LA levels. In addition, the highly crystalline films exhibited low *T*_d_ values. Reducing the L-LA content of the copolymer evidently decreased the degree of crystallinity, which in turn increased the haze value. This change in crystallinity was expected to increase the incorporation of essential oil into the copolymer.

**Table 2:** Physical and morphological properties of the poly(L-LA-*ran*-VL) films.

Comp.^a^ [mol/mol]	*M*_n_/10^4b^ [g/mol]	*X*_c XRD_^c^ [%]	Haze^d^ [%]	*T*_t_^d^ [%]	*T*_p_^d^ [%]	*T*_d_^d^ [%]
91/9	6.48	25.3	58.0	91.1	38.3	52.9
83/17	5.13	23.9	76.4	93.9	22.2	71.7
73/27	6.62	23.4	82.0	94.3	17.0	77.3

^a^Determined by ^1^H NMR. Comp.: Composition. ^b^Determined by GPC. ^c^Determined by XRD. ^d^Determined by haze meter.

**Table 3:** Physical and morphological properties of the poly(L-LA) (LACEA H-100) and poly(VL) films.

Film	*M*_n_/10^4a^ [g/mol]	*M*_w_/*M*_n_^a^	*T*_m_^b^ [°C]	Δ*H*_m_^b^ [J/g]	*T*_g_^b^ [°C]	*X*_c XRD_^c^ [%]	Haze^d^ [%]	*T*_t_^d^ [%]	*T*_p_^d^ [%]	*T*_d_^d^ [%]
PLLA	8.96	1.64	170.4	34.9	60.6	7.2	26.1	90.5	66.9	23.6
PVL	6.31	1.58	59.7	96.1	−57.7	27.1	98.6	67.7	0.9	66.7

^a^Determined by GPC. ^b^Determined by DSC. ^c^Determined by XRD. ^d^Determined by haze meter.

**Figure 7:**
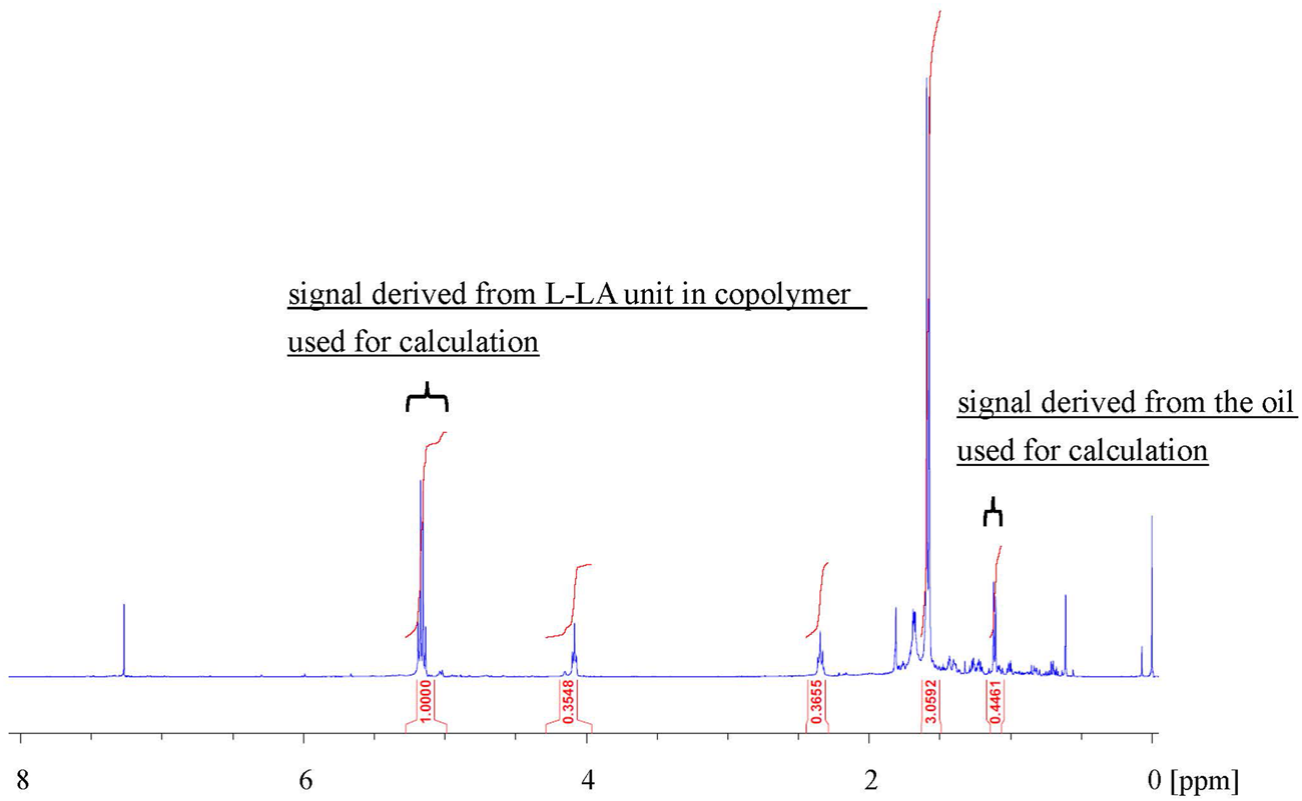
^1^H NMR spectrum of poly(L-LA-*ran*-VL) (73/27) after impregnation with essential bark oil using scCO_2_.

**Figure 8:**
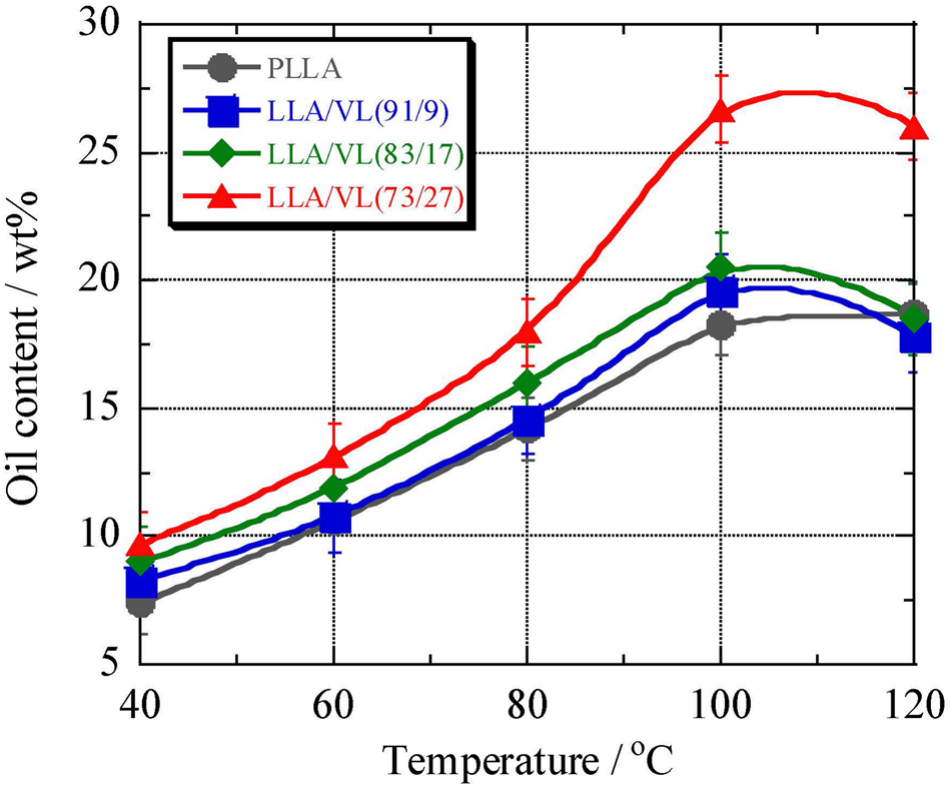
Essential oil impregnation of L-LA/VL random copolymer films (using scCO_2_ at 14 MPa for 3 h) as function of temperature.

To compare the level of incorporation of the essential bark oil in the various poly(L-LA) random copolymers using scCO_2_, impregnation experiments were conducted at temperatures from 40 to 120 °C and at 14 MPa for 3 h. During these trials, the temperature was reduced to approximately 40 °C after processing for 3 h, the pressure was gradually released, and the sample was taken out from the reaction vessel. Because some CO_2_ was retained in the copolymer films after processing, the oil content was determined by ^1^H NMR (Fig. 7), and the results are shown in Fig. 8. The oil content in each specimen was calculated using a calibration curve prepared based on the solution which precisely weighing the polymer and the essential oil and dissolved them. The calibration curve was generated by plotting the ratio of the integrated area of the peak derived from the CH group in L-LA unit to that of the peak due to the oil in the ^1^H NMR spectra against the known oil content. The lowest level of oil (7.4%) was obtained at 40 °C and 14 MPa and this value increased along with the temperature, giving oil concentrations of 18.5% at both 100 and 120 °C. Because poly(L-LA) dissolves in scCO_2_ above 120 °C, this temperature should allow the maximum incorporation of the essential oil into the polymer. In a previous study, we determined that synthetic copolymers of L-LA with CL or TEMC could hold more oil than poly(L-LA)^24^. However, these materials had a tendency to dissolve above 80 °C, and so were not suitable for practical applications. In this work, copolymers of L-LA with VL were used and the poly(L-LA-*ran*-VL) samples (91/9-73/27) did not dissolve up to 120 °C. In fact, this material was found to have a *T*_m_ as high as 160 °C, as shown in Table 1. In addition, since the Δ*H*_m_ was greater than 25 J/g, it was predicted that this polymer would not dissolve during scCO_2_ processing.

In the case of the poly(L-LA-*ran*-VL), the copolymer having the composition ratio 91/9 showed almost the same oil content as the pure poly(L-LA), and had a slightly higher oil content at 100 °C. The 83/17 specimen held more oil than the 91/9 sample over the entire temperature range. The *T*_m_ and Δ*H*_m_ values for the 83/17 sample were almost equal to those for the 91/9 sample and the poly(L-LA). However, because the *T*_g_ for this material was lower, the oil content of the copolymer was only slightly increased at all temperatures. Furthermore, the impregnation of the 73/27 material was significantly improved, especially at 100 and 120 °C. The oil concentration in all the poly(L-LA-*ran*-VL) samples at 120 °C was lower than at 100 °C, and the data for the copolymers also differed from that for the poly(L-LA) at this temperature. These results are attributed to the *T*_g_ values for the copolymers (which were 50 °C or less) in contrast to the *T*_g_ for poly(L-LA), which is over 60 °C.

The incorporation of essential oil into the poly(L-LA-*ran*-VL) and into the other L-LA copolymers made previously was also compared. In previous work, L-LA was copolymerized with CL, TEMC or DXO, to give poly(L-LA-*ran*-CL), poly(L-LA-*ran*-TEMC) or poly(L-LA-*ran*-DXO). The poly(L-LA-*ran*-CL) and poly(L-LA-*ran*-DXO) had very similar thermal properties, although their *T*_m_ and Δ*H*_m_ were lower than those for poly(L-LA-*ran*-VL) at the same composition ratios, while the values for the poly(L-LA-*ran*-TEMC) were even lower. The poly(L-LA-*ran*-VL) appears to have had a greater degree of crystallinity, based on its higher *T*_m_. Because the poly(L-LA-*ran*-VL) was more difficult to dissolve in scCO_2_ compared to the other copolymers, it would be expected to hold less essential oil. The result of impregnation trials using the four types of copolymers at a 9/1 composition ratio were compared. All these results, except those for the poly(L-LA-*ran*-VL) (91/9), have been previously reported^24^. The oil content of the poly(L-LA) was 7.4% at 14 MPa and 40 °C and the oil concentration increased along with the processing temperature, to 18.3% at 100 °C. Although the poly(L-LA-*ran*-CL) (88/12) and poly(L-LA-*ran*-DXO) (92/8) had almost the same *T*_m_ values, because the Δ*H*_m_ for the poly(L-LA-*ran*-DXO) was higher than that for the poly(L-LA-*ran*-CL), the difference in the oil content of these two materials increased as the temperature was increased, to a maximum ratio of 1.7 at 100 °C. Because the *T*_m_ and Δ*H*_m_ values for the poly(L-LA-*ran*-TEMC) (89/11) were lower than those for the poly(L-LA-*ran*-CL) (88/12), the oil content was greater even at a low processing temperature (for example, 29% at 80 °C), although the film dissolved at 100 °C. The poly(L-LA-*ran*-VL) had a very similar Δ*H*_m_ value to the poly(L-LA-*ran*-DXO) because the *T*_m_ for the former (171.3 °C) was 16 °C higher than that for the latter. Thus, the essential oil was not readily incorporated from the film surface and there was little increase in the oil concentration at high temperatures. The data for the poly(L-LA) and poly(L-LA-*ran*-DXO) (92/8), which had higher *T*_m_ and Δ*H*_m_ values, show that increasing the temperature slowly raised the oil levels in the polymers, to 20% at 100 °C, although this value was lower than that obtained for the poly(L-LA-*ran*-DXO) (92/8).

Next, the results obtained from the impregnation of various copolymers having an 8/2 composition ratio were compared. It has been previously reported that the oil content of poly(L-LA-*ran*-TEMC) (79/21) and poly(L-LA-*ran*-CL) (83/17) was somewhat higher at any given temperature compared with copolymers having a 9/1 composition ratio, and that the impregnation of poly(L-LA-*ran*-DXO) (82/18) was increased remarkably at 100 °C and 14 MPa. Because the poly(L-LA-*ran*-TEMC) (79/21) had a low *T*_m_ of 107.4 °C and Δ*H*_m_ of 8.2 J/g, the surface of the film was already dissolved in the scCO_2_ at 40 °C and 14 MPa. Since the poly(L-LA-*ran*-VL) (91/9) had similar *T*_m_ and Δ*H*_m_ values to the poly(L-LA-*ran*-VL) (83/17), the oil concentrations were also similar at each temperature.

In the case of the copolymers having 7/3 composition ratios, since the *T*_m_ and Δ*H*_m_ of the poly(L-LA-*ran*-CL) (72/28), poly(L-LA-*ran*-TEMC) (76/24) and poly(L-LA-*ran*-DXO) (75/25) were significantly lower than those of the poly(L-LA), these copolymers melted at 80 and 100 °C. It has been previously reported that copolymers having a 7/3 composition ratio are readily impregnated with oil. Incorporation of the oil into the poly(L-LA-*ran*-VL) (73/27) increased over the entire temperature range, and was 1.3 times greater than the amount contained in the poly(L-LA-*ran*-VL) (83/17) at 100 °C. These results are ascribed to the lower *T*_m_ (by 5 °C) of the poly(L-LA-*ran*-VL) (73/27) compared to the 83/17 specimen. Because the *T*_m_ and Δ*H*_m_ values for the poly(L-LA-*ran*-VL) were higher than those of the other copolymers, this material was not plasticized as well during scCO_2_ processing, and so showed a lower oil uptake. Overall, the poly(L-LA-*ran*-VL) appears most suitable for this purpose because it has a higher L-LA proportion and exhibits minimal dissolution.

**Figure 9:**
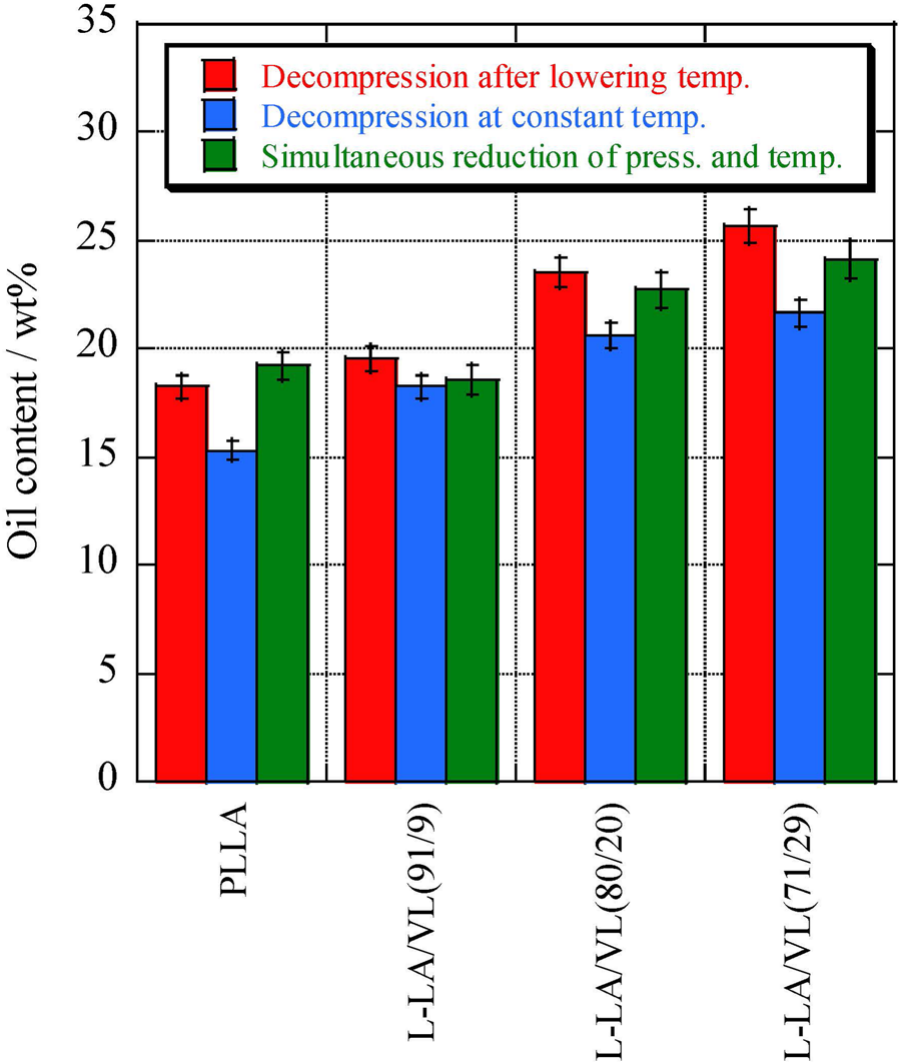
Amount of essential oil incorporated into L-LA/VL random copolymer films using different scCO_2_ processing parameters.

The impregnation trials described above involved lowering the temperature to 40 °C after processing for 3 h, following by decompression to atmospheric pressure and removal of the sample [termed method (1)]. Impregnation of the essential oil followed by the release of pressure at a constant temperature is referred to herein as method (2), while the simultaneous release of pressure and cooling is method (3). These trials were performed at 14 MPa and 100 °C for 3 h, and the results from all three methods compared. The results of the different scCO_2_ impregnation approaches are summarized in Fig. 9, where methods (1), (2) and (3) are indicated by black, mesh and gray bars, respectively. Method (2) evidently lowered the oil content by 1.3 to 4.0% relative to method (1). The release of the essential oil from the film was evidently increased by melting of the sample during decompression at constant temperature, thus decreasing the oil content. However, the oil content of the poly(L-LA-*ran*-VL) (91/9) was almost equivalent when using methods (1) and (2). This copolymer had the highest *T*_m_ and Δ*H*_m_ among the films used for the experiments, and it can be presumed that the degree of softening was minimal in the supercritical state at 100 °C. Although the oil content obtained using method (3) was 0.3 to 4.4% higher than for method (2) in each case, there were only minimal differences compared to method (1). Reducing the pressure and temperature simultaneously [method (3)] avoids softening of the film in response to lowering the temperature, and so the resulting oil content was equivalent to that obtained from method (1). Overall, these results show that method (1), consisting of decompressing after reducing the temperature to 40 °C, gave the highest oil concentration.

### Enzymatic degradation and controlled vapor release during hydrolysis of poly(L-LA-*ran*-*δ*-valerolactone)

**Figure 10:**
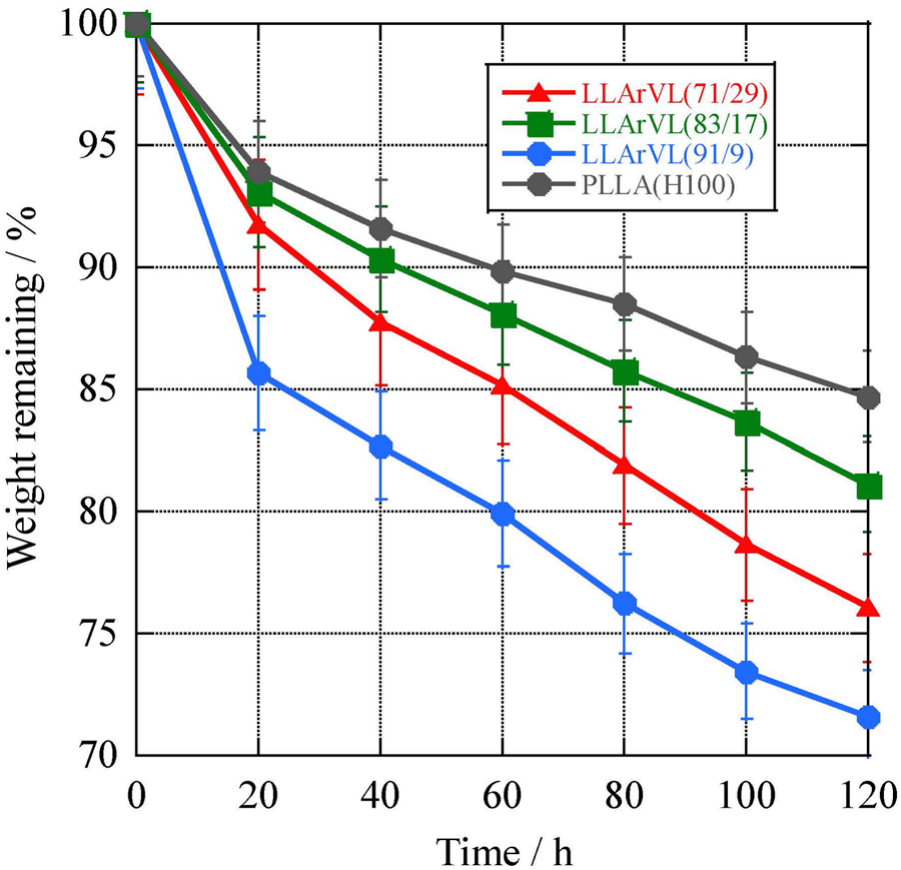
Enzymatic degradation of poly(L-LA-*ran*-VL) or poly(L-LA) with proteinase K in a Tricine buffer at pH 8.0.

In order to use the synthetic poly(L-LA-*ran*-VL) as a base material for the controlled release of a repellent, the degradability of the polymer needs to be evaluated. For this reason, enzymatic degradability tests were performed with proteinase K (PTK), using 100 μm thick films prepared by the solvent-casting method. The results are presented in Fig. 10. The degradation of LACEA H-100 was also assessed for comparison purposes. The degradation of the poly(L-LA) was slow, with only 85% degraded after 120 h. The sample with 9 mol% VL showed the most rapid degradation rate among the polymers used, with 72% degradation at 120 h. Higher VL proportions reduced the degradation rate, suggesting that the VL units are not easily degraded with PTK. However, the 83/17 copolymer showed slower degradation than the 71/29 specimen, and this result is attributed to the higher crystallinity of the copolymer, which had 17 mol% VL.

**Figure 11:**
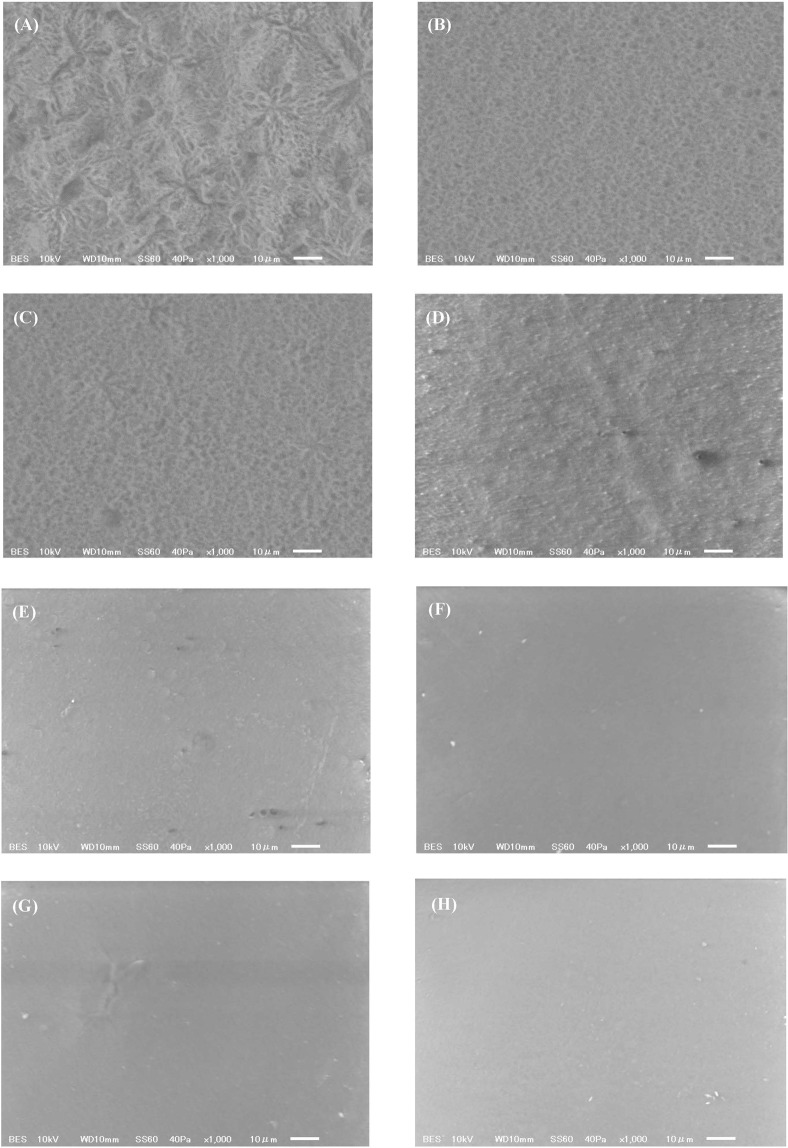
SEM images of poly(L-LA-*ran*-VL) samples after degradation by proteinase K over 120 h, for samples with composition (**A**) 71/29 (mass loss 76%), (**B**) 83/17 (mass loss 81%) and (**C**) 91/9 (mass loss 72%) and of (**D**) 100/0 (LACEA H-100) (mass loss 85%) degraded under same conditions. SEM images before degradation of poly(L-LA-*ran*-VL) samples with composition (**E**) 71/29, (**F**) 83/17 and (**G**) 91/9 and of (**H**) 100/0 (LACEA H-100).

To further examine changes in the degradability of poly(L-LA-*ran*-VL) specimens with PTK, scanning electron microscopy (SEM) images were obtained of samples that had been degraded by approximately 80% (Fig. 11). The samples before degradation were found to have a very smooth surface (data not shown). In contrast, the SEM image of poly(L-LA-*ran*-VL) (71/29) shows changes following enzymatic degradation by PTK at 120 h, with an accompanying 76% mass loss [Fig. 11(A)]. Small cavities less than 10 mm in diameter appeared in the copolymer, and these cavities became smaller with increasing L-LA content. In the case of the copolymer with an L-LA content greater than 80%, cavities less than 5 mm in diameter were observed.

**Figure 12:**
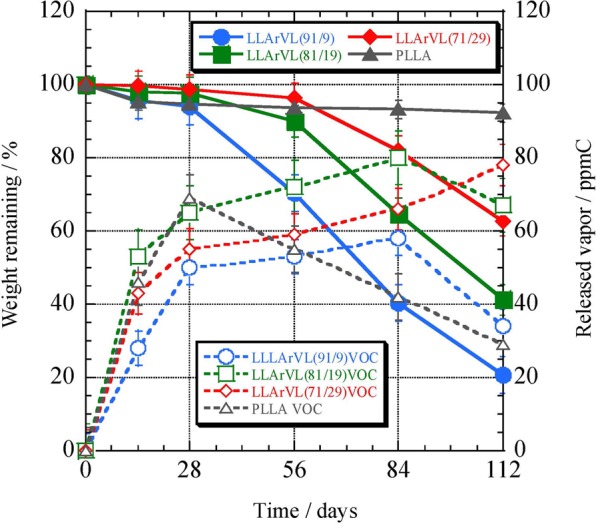
Mass loss and oil vapor release during the hydrolysis of poly(L-LA-*ran*-VL) or poly(L-LA) in a phosphate buffer at pH 7.0.

The release of the oil from the copolymers in the form of vapor during degradation was monitored using a volatile organic compound analyzer. During these trials, the hydrolysis of poly(L-LA-*ran*-VL) samples impregnated with oil was conducted in a phosphate buffer (pH 7.0) at 37 °C, with the results shown in Fig. 12. Here, the solid and dashed lines indicate the degree of hydrolysis (%) of the copolymer and the vapor release (ppm C). The poly(L-LA-*ran*-VL) specimens contained the following oil levels: 91/9 = 19.7%, 81/19 = 24.9%, 71/29 = 30.3%. The pure poly(L-LA) contained 15.8% oil. The physical properties of the poly(L-LA) synthesized for the gas release experiments were: *M*_n_ = 7.31 × 10^4^ g/mol, *M*_w_/*M*_n_ = 1.63, *T*_m_ = 177.2 °C, Δ*H*_m_ = 54.1 J/g and *T*_g_ = 59.5 °C. The poly(L-LA) was hydrolyzed very slowly, with only 8% mass loss after 112 days. However, the vapor release from this material showed a maximum value of 69 ppm C at only 28 days, and decreased in proportion to the amount of time elapsed after that point. The *T*_m_ and Δ*H*_m_ values for the poly(L-LA) were both relatively high, and so high levels of the essential oil could not be incorporated. As a result, it is believed that only the areas near the film surface contained the oil.

The poly(L-LA-*ran*-VL) with a higher L-LA content was hydrolyzed sooner than the other copolymers, such that the poly(L-LA-*ran*-VL) with a 91/9 composition ratio was 21% hydrolyzed after 112 days. However, the vapor release from this specimen was the lowest, with a maximum of 58 ppm C at 84 days. Since the oil content of the poly(L-LA-*ran*-VL) (91/9) was the lowest, even rapid hydrolysis led to a minimal vapor release. Although hydrolysis of the 81/19 specimen occurred sooner than that of the 91/9 material, the vapor release was highest during the early stages of hydrolysis, with a maximum of 80 ppm C at 84 days. This early release of the vapor even in the case of a high oil concentration is ascribed to the rapid hydrolysis of the sample. Although hydrolysis of the 71/29 sample was the slowest, with 63% remaining at 112 days, the vapor release continually increased up to 112 days because of the high oil content, giving a maximum of 78 ppm C at 112 days.

These vapor release data show that the poly(L-LA-*ran*-VL) (91/9), having the lowest oil content, underwent early hydrolysis of the base material and its release of vapor changed very little during the hydrolysis. On the contrary, the poly(L-LA-*ran*-VL) (71/29) had a high oil content but exhibited slow hydrolysis and generated more vapor in conjunction with a continuous increase during the hydrolysis experiment. Because the oil levels in the highly crystalline polymers were low, even rapid hydrolysis did not result in a large release of vapor. Furthermore, if both the *T*_m_ and the crystallinity are low, the oil content is increased but the slower hydrolysis of the base material enables vapor release over a long period of time.

## Experimental Section

### Materials

L-LA [(3*S*)-*cis*-3,6-dimethyl-1,4-dioxane-2,5-dione] (Aldrich) was purified by recrystallization from tetrahydrofuran. VL (Wako Pure Chemicals Ind., Ltd.) was employed as a monomer for copolymerization with L-LA and was used as-received without purification. Sn(oct)_2_ (Sigma) was used as a catalyst, also without purification. Methanol and chloroform were purchased from the Japan Alcohol Trading Co., Ltd. and the Tokuyama Corporation, respectively. Benzene and ethylbenzene were purchased from Wako Pure Chemicals Ind., Ltd., dried over CaH_2_, and then filtered before use. Poly(L-lactide) (LACEA H-100) was donated by Mitsui Chemicals, Inc. Films of the synthetic copolymers were prepared by the solvent-casting method. In this process, a solution prepared by dissolving 1000 mg of the copolymer in 15 ml chloroform was poured into a Teflon dish with a diameter of 100 mm and placed in a fume hood, after which the solvent was allowed to slowly evaporate at approximately 25 °C. The thickness of the resulting film was measured using a micrometer (MDC-25MJ, Mitutoyo Corporation). Essential bark oil from *Thujopsis dolabrata* var. *hondae* (herein referred to simply as essential oil) was purchased from Sugiyama Mokuzai K.K. (Aomori, Japan) and used without purification. This material contained both neutral and acid oil. The majority of the former comprised the sesquiterpene *Thujopsis dolabrata* var. *hondae* (also known as thujopsene), which the latter contained substances such as hinokitiol and *β*-dolabrin (which have significant antibacterial properties) at a level of 2%. Proteinase K from *Tritirachium album* and *N*-[tris(hydroxymethyl)methyl]-glycine (Tricine) (for use as a pH 8.0 buffer) were purchased from Nacalai Tesque, Inc. and used at 37 °C. Ion-exchanged water was employed during all degradation tests. The sodium hydroxide used for pH adjustment was purchased from Wako Pure Chem. Ind., Ltd.

### Characterization of polymers

The number- and weight-average molecular weights of the synthetic copolymers were determined by gel permeation chromatography (GPC) using a Hitachi D*-*2520 chromatograph equipped with a Shodex GPC K-804 column, employing chloroform as the mobile phase at 40 °C and a flow rate of 1.0 mL/min in conjunction with a refractive index detector. The molecular weights were calculated by comparison with a polystyrene standard.

Thermal characteristics [melting point (*T*_m_), glass transition point (*T*_g_) and enthalpy of melting (Δ*H*_m_)] were obtained using differential scanning calorimetry (DSC) using a Rigaku Thermo Plus 2/DSC8230 instrument. In these trials, samples (5 mg) were heated at a rate of 10 °C/min from –20 to 200 °C in a nitrogen stream. The *T*_m_ and Δ*H*_m_ values were determined during the initial heating step, while the *T*_g_ value was determined during the second heating step.

The proportion of L-LA in each specimen was determined by ^1^H nuclear magnetic resonance (^1^H NMR) spectroscopy using a BRUKER AVANCE III HD 400 spectrometer. The amount of essential oil incorporated into the copolymer was also found using ^1^H NMR spectroscopy. Chemical shifts are reported herein relative to the residual chloroform signal at 7.27 ppm.

During the oil incorporation experiments, the percent crystallinity (*Χ*_c XRD_) and the haze values for the synthetic copolymer films were assessed to examine the effect of crystallinity on the oil content of the films. Crystal structures were investigated with a Rigaku Rint 2100X-ray diffractometer equipped with a scintillation counter, employing CuKα radiation (30 kV, 15 mA) over a scan region of 2*θ* = 8 to 35° and at a scan rate of 2 °/min. Poly(L-LA-*ran*-VL) specimens were each assessed three times and the average values are reported. The *Χ*_c XRD_ for each copolymer film sample was calculated based on the equation^32^

$${X}_{cXRD}( \% )=\frac{{\int }_{2{\theta }_{1}}^{2{\theta }_{2}}\,{I}_{c}(2\theta )d(2\theta )}{{\int }_{2{\theta }_{1}}^{2{\theta }_{2}}\,I(2\theta )d(2\theta )}\times 100$$

Each film sample was scanned at 8° (2*θ*_1_) and 35° (2*θ*_2_), and *I*_c_(2*θ*) and *I*(2*θ*) are the diffraction intensities obtained from both scans, representing crystalline and noncrystalline domains, respectively.

The haze values for the films were determined using an NDH 4000 haze meter (Nippon Denshoku Industries Co., Ltd.), measuring the total light transmittance (*T*_t_ = *T*_p_ + *T*_d_), parallel light transmittance (*T*_p_) and diffuse transmittance (*T*_d_) simultaneously. The haze value was determined using the equation

$$Haze\,( \% )=\frac{diffuse\,{transmittance}\,({T}_{d})}{total\,light\,{transmittance}\,({T}_{t})}\times 100$$

The haze, *T*_t_, *T*_p_ and *T*_d_ values reported herein represent the averages of triplicate analyses.

### Synthesis of poly(L-lactide-*ran*-*δ*-valerolactone)

Poly(L-LA-*ran*-VL) was produced using an efficient one-pot synthesis reported previously^33^. This process consists of continuous dehydration of the monomers and solvent with subsequent polymerization. The copolymer was synthesized using Sn(oct)_2_ (0.1 mol% relative to the amount of monomer) as the catalyst at 150 °C over a time span of 24 h, employing an oil bath for heating. Benzene was added at a ratio of 10 ml per g monomer as the azeotropic distillation solvent and ethyl benzene was added at a ratio of 4 ml per g monomer as the polymerization solvent. Azeotropic distillation was initially conducted for 30 min at approximately 110 °C to remove trace amounts of moisture from the reaction mixture, after which the temperature was raised to 150 °C and the polymerization was performed for 24 h. This innovative polymerization method is characterized by its ability to perform purification of both monomers and polymerization using the same distillation equipment, representing an efficient one-pot process. In this process, an azeotropic mixture is necessary to remove water in the monomers in order to successfully perform the subsequent polymerization. The system can easily and effectively utilize general distillation equipment or a distillation tower. The resulting mixture was dissolved in chloroform and then added to an excess of methanol to precipitate the polymer. The product was subsequently dried under reduced pressure at 60 °C and the molecular weights, compositions and thermal properties of the various copolymers were determined by GPC, ^1^H NMR and DSC, respectively.

### Incorporation of essential bark oil from *Thujopsis dolabrata* var. *hondae* into poly(L-LA-*ran*-*δ*-valerolactone) using supercritical carbon dioxide

Incorporation of essential bark oil into the poly(L-LA-*ran*-VL) using supercritical carbon dioxide was conducted according to a previously reported method^24,33,34^. Poly(L-LA-*ran*-VL) was used in the form of 100 μm thick films prepared by the solvent-casting method. Impregnation into the L-LA random copolymer film (ca. 100 mg) was carried out using essential bark oil of 2 g inside a pressure-resistant container made from stainless steel (volume: 0.5 L). The processing was performed with stirring at 100 rpm under scCO_2_ at 14 MPa for 3 h, using a high-pressure reaction apparatus (MMJ-500, OM Lab-Tech Co., Ltd.) equipped with a non-pulsating flow pump (NP-KX-500, Nihon Seimitsu Kagaku Co., Ltd.). Five films could be simultaneously processed in this apparatus. The temperature was varied over the range of 40 to 120 °C at a constant pressure of 14 MPa and using a constant reaction time of 3 h. The scCO_2_ was prepared by first heating the container to a temperature higher than the critical temperature of 31.1 °C, after which the atmosphere inside the container was replaced with CO_2_ gas three times. The container was then filled with CO_2_ using a pump intended for use with liquid. To accomplish this, a coolant flow was used to lower the pump head temperature to approximately 0 °C, after which the system pressure was increased to the desired value simply by compressing the liquid CO_2_. The liquid CO_2_ that exited the pump changed to either a gas or supercritical fluid depending on the temperature and pressure of the stainless steel tube connecting the pump to the container. The liquefied CO_2_ passing through the pump was turned into gas or supercritical fluid within the stainless steel tube heated to about 40 °C. Because the CO_2_ temperature increased upon compression by the pumping apparatus, the temperature of the container was kept somewhat lower than the actual desired temperature during filling of the chamber with CO_2_. After filling, the temperature and pressure were adjusted to the desired values. After processing, the polymer was cooled to 40 °C and the pressure in the vessel was lowered gradually over a period of 2 h, after which the sample was removed from the container and weighed. In other trials, supercritical fluid impregnation of the oil was carried out at 14 MPa and 100 °C for 3 h followed by either decompression at a constant temperature or reduction of the pressure and temperature simultaneously. Because the samples contained high concentrations of CO_2_ following impregnation, the essential oil levels were determined by ^1^H NMR spectroscopy.

### Degradation and controlled release of poly(L-LA-*ran*-*δ*-valerolactone)

Proteinase K from *Tritirachium album* (activity: 40.1 IU/mg) was used as the enzyme for the degradation of poly(L-LA-*ran*-VL) samples. In these trials, the enzyme was dissolved in a 50 mM pH 8.0 Tricine buffer at a level of 1 IU/mg polymer in a 50 mL sample bottle. The bottle was then placed in a shaking water bath for 15 min until it reached the degradation temperature of 37 °C. A polymer film with a mass of approximately 30 mg was prepared by the solvent-casting method and sealed in a mesh bag made from polyethylene sheets (1 mm × 1 mm mesh), then placed in the bottle containing the enzyme and buffer. The enzymatic degradation tests were conducted at 37 °C with shaking at 100 rpm. The enzyme and buffer solutions were replaced every 40 h to maintain enzymatic activity at the desired level throughout the experiments. After incubation, the samples were washed thoroughly with deionized water and dried under vacuum for 3 h. Degradability was evaluated based on the remaining sample mass.

Hydrolysis trials were performed in a 50 mM pH 7.0 phosphate buffer to evaluate the release rates of the essential bark oil vapor, using poly(L-LA-*ran*-VL) films impregnated with essential oil. Poly(L-LA) and poly(L-LA-*ran*-VL) specimens having monomer ratios of 91/9, 81/19 and 71/29 were impregnated with the oil via scCO_2_ processing in preparation for these trials, and the oil concentrations in these samples were determined to be 15.8, 19.7, 24.9 and 30.3%, respectively. In each trial, a polymer sample with a mass of approximately 1200 mg and the buffer solution were transferred into a 1 L polyvinyl fluoride (PVF) bag equipped with a two-way valve, after which the bag was filled with synthetic air and hydrolysis was performed at 37 °C in an incubator. A 200 mL aliquot of the air in the bag was sampled at 28 day intervals and the amount of essential oil vapor released from each copolymer sample was determined using a volatile organic compound analyzer (VMS-1000F, Shimadzu Corp.). Following each sampling, all the air was discharged from the bag and it was refilled with fresh synthetic air to the original volume of 1 L. Degradation and controlled release tests were conducted three times and the average values are reported.

## Conclusions

scCO_2_ processing at 14 MPa and 60 °C or above requires the preparation of an L-LA copolymer having a film surface that does not dissolve, thus allowing it to hold the essential oil. In this work, a copolymer made of L-LA and VL and having high *T*_m_ and *T*_g_ values was synthesized as the base polymer for a controlled release material, then impregnated with essential bark oil from *Thujopsis dolabrata* var. *hondae*. A copolymer with a high degree of polymerization was obtained using an efficient one-pot process. This technique produced poly(L-LA-*ran*-VL) with a high molecular weight at high yield. The *T*_m_ and Δ*H*_m_ of the poly(L-LA-*ran*-VL) were higher than those of poly(L-LA-*ran*-CL), and the copolymer film did not dissolve during scCO_2_ processing even at 14 MPa and 120 °C. Thus, poly(L-LA-*ran*-VL) is superior to other copolymers, such as poly(L-LA-*ran*-CL), during scCO_2_ processing.

Impregnation experiments with the essential oil using scCO_2_ showed that films of poly(L-LA-*ran*-VL) (91/9 to 73/27) did not dissolve up to 120 °C. Poly(L-LA-*ran*-VL) (91/9) absorbed oil to almost the same level as poly(L-LA), and actually had a slightly higher oil content at 100 °C. Poly(L-LA-*ran*-VL) (83/17) also held somewhat more oil than poly(L-LA-*ran*-VL) (91/9) at all temperatures. The oil content of the poly(L-LA-*ran*-VL) (73/27) was much higher than those of the other specimens, with especially high oil concentrations at 100 and 120 °C.

The optimal method of adding the oil was determined using three methods: decompression after reducing the temperature [method (1)], decompression at constant temperature [method (2)], and simultaneous reduction of pressure and temperature [method (3)]. The highest essential oil content was obtained using method (1), while methods (2) and (3) gave increasingly lower levels of oil impregnation.

During hydrolysis using a phosphate buffer solution at pH 7.0, the oil vapor released upon degradation of the copolymer was measured. Although the poly(L-LA-*ran*-VL) (91/9) had the lowest oil concentration and underwent early degradation of the base copolymer, it exhibited the least change in vapor release during hydrolysis. Conversely, because the poly(L-LA-*ran*-VL) (71/29) degraded slowly and held more oil, the vapor release was slightly greater, with a continual increase throughout the hydrolysis.
